# Key Multimodal Fundus Imaging Findings to Recognize Multifocal Choroiditis in Patients With Pathological Myopia

**DOI:** 10.3389/fmed.2021.831764

**Published:** 2022-01-24

**Authors:** Roberto Gallego-Pinazo, Sara Hernández, Rosa Dolz-Marco

**Affiliations:** ^1^Unit of Macula, Clinica Oftalvist, Valencia, Spain; ^2^Department of Ophthalmology, Hospital 12 de Octubre, Madrid, Spain

**Keywords:** multifocal choroiditis, myopia, optical coherence tomography, optical coherence tomography (angiography) (OCTA), chorioretinal atrophy, fundus autofluorescence, inflammatory choroidal neovascularization, Punctate Inner Choroidopathy (PIC)

## Abstract

Myopia represents a major socioeconomic burden with an increasing prevalence worldwide. Pathologic myopia refers to myopic patients with structural changes in the posterior pole including different patterns of chorioretinal atrophy, choroidal neovascularization (CNV) and vitreomacular tractional diseases. Multifocal choroiditis (MFC) is one of the most frequent noninfectious posterior uveitis, and epidemiologically typically affects young myopic females. Acute and chronic chorioretinal atrophic changes are the hallmark feature of MFC, with CNV developing in almost one third of cases. Thus, differentiation of inflammatory lesions due to MFC or neurodenegerative lesions due to pathologic myopic is key in order to establish a particular prognosis, follow-up schedule, and therapeutic approach. The aim of the present manuscript is to summarize and illustrate the main multimodal imaging features of these diseases.

## Introduction

Myopia is a medical condition characterized by blurred distance vision because of images of distant objects focusing in front of the retina, what is mostly due to excessive elongation of the eye. The sociosanitary impact of myopia is increasing worldwide with an estimated overall prevalence of 2.5 billion cases, comprising 10–30 % in the adult population in many countries and 80–90% in young adults in some parts or East and Southest Asia (1, 2).

The term pathologic myopia is usually used to describe patients with myopia and structural changes in the posterior pole (3), including vitreoretinal tractional changes, choroidal neovascularization (CNV) and chorioretinal atrophy (2, 4, 5).

Focusing on chorioretinal atrophic changes related to pathologic myopica we can divide them into fundus tessellation, diffuse chorioretinal atrophy, and patchy chorioretinal atrophy (PCA). PCA can be observed as whitish well-defined lesions of various shapes and configurations, probably related to complete closure of the choriocapillaris. PCA may develop from lacquer cracks (Lc), within areas of advanced diffuse chorioretinal atrophy, and along the border of posterior staphyloma (6, 7).

Idiopathic Multifocal Choroiditis (MFC) and Punctate Inner Choroidopathy share clinical, structural and prognostic insights. Thus, both terms are generally used indistinctly and a consensus to differentiate both entities remains undetermined. They represent a form of non-infectious posterior uveitis characterized by a chronic, recurrent and usually bilateral disease (8–10). Acute inflammatory lesions appear as single or multiple yellow-grayish spots that may evolve progressively into punched-out atrophic scars, with a variable degree of pigmentation. Also, MFC may eventually associate inflammatory choroidal neovascularization in about one third of cases through the follow-up (8, 11–13).

Thus, the ability of clinicians to differentiate findings suitable for pathologic myopia or MFC is key to provide an accurate prognosis and follow-up plan for patients, and thus to consider the addition of immunosuppressive therapy in cases with visual threatening atrophic and neovascular changes. The aim of the present manuscript is to summarize the key multimodal imaging findings in cases with MFC and the main helpful tips to differentiate these from those of PCA and myopic CNV.

## Chorioretinal Atrophic Changes

**Figure 1:**
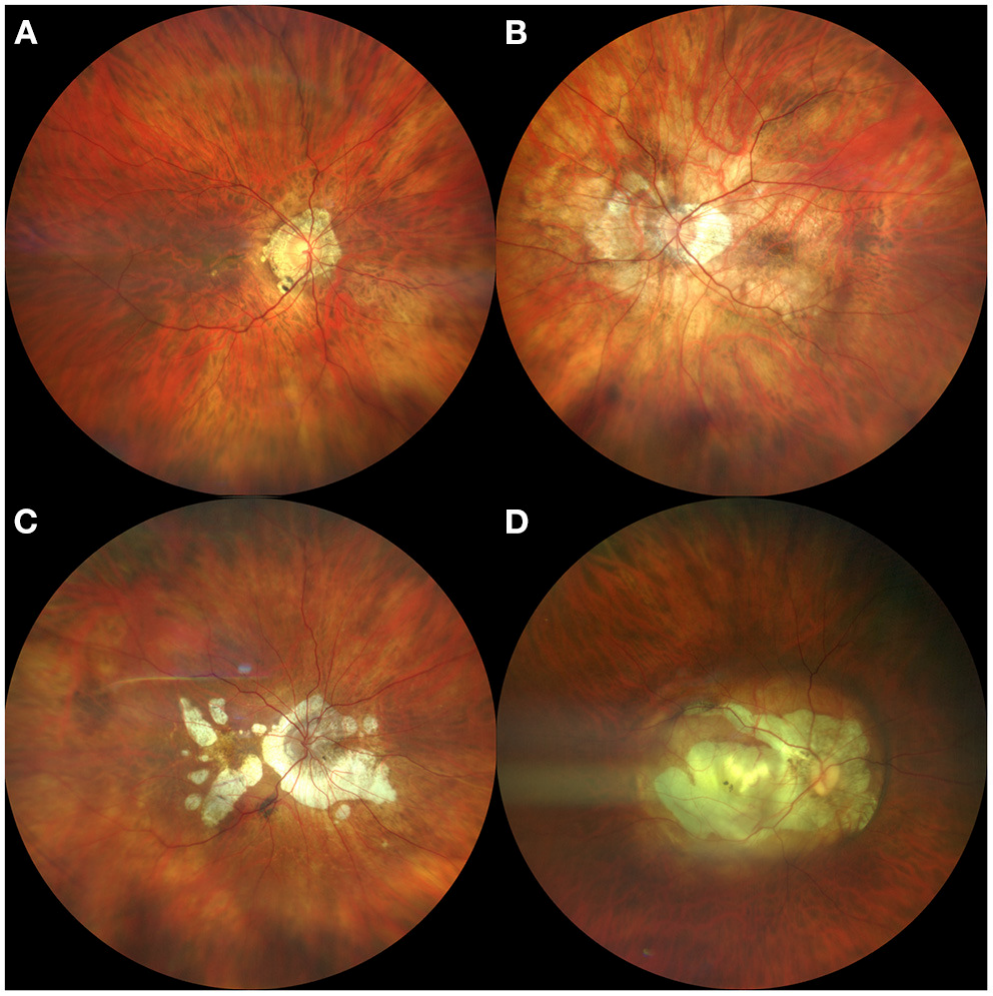
Summary of different myopic atrophic retinal changes. **(A)** Fundus tessellation; **(B)** Diffuse chorioretinal atrophy; **(C)** Patchy chorioretinal atrophy; **(D)** Complete macular atrophy.

Ruiz-Medrano et al recently published a simplified classification of retinal atrophic patterns associated with high myopia (2). The authors define category A0 in the absence of any particular atrophic change in the fundus examination; category A1 for cases with fundus tessellation; category A2 for cases with diffuse chorioretinal atrophy; category A3 for patients with PCA; and category A4 for cases with complete macular atrophy (Figure 1).

The differentiation of PCA due to pathologic myopia, and chorioretinal atrophic lesions due to MFC may be challenging. However, this should be carefully addressed by physicians given the relevant prognostic implications of the origin of atrophic lesions.

PCA consists in well-defined, grayish white lesion(s), mainly localized within the macular area and around the optic disk. The size may vary between one or several choroidal lobules (14). The appearance of myopic PCA lesions developed within areas of diffuse chorioretinal atrophy is usually elliptical, whereas those developed from Lc exhibit oval configurations; on the other hand, PCA related to posterior staphyloma develops along the edge of this posterior wall morphologic change (6, 15, 16). In stereoscopic fundus image, is possible to appreciate the excavation of the area compared to surrounding diffuse atrophy, due to the complete loss of choriocapillaris. Sometimes there is pigment clumping, especially along the margin or along large choroidal vessels that are often visible within the area of PCA. In advanced cases it is possible to observe the sclera and even the retrobulbar vessels by transparence (14). OCT reveals disruption of outer retinal layers and retinal pigment epithelium (RPE); therefore, choroidal hypertransmission is the tomographic hallmark feature of RPE atrophy. PCA appears typically hypoautofluorescent on fundus autofluorescence (FAF) images, and early hyperfluorescent (window defect) on fluorescein angiography (FA) images (14).

Mechanical fissures in the retinal pigment epithelium and Bruchs's membrane-choricapillaris complex lead to the formation of Lc; these can be observed as fine, irregular, yellowish longitudinal lines, often branching and crisscrossing located mostly on the posterior pole (radiating from the optic disc, at the papillomacular bundle, through and around the macula), but their usually difficult detection depends on their stage and the surrounding atrophy (14, 17, 18). Lc are usually too narrow to be appreciated on OCT, but macular choroidal thinning is frequently observed. ICGA is the best approach to detect Lc, showing linear hypofluorescence in the late phase. On FA, they are hyperfluorescent due to window defect by the atrophic overlying RPE, but the latter develops months after the onset of the rupture, being difficult to demonstrate LC at their early stages. Presumably, this fact could be explained by the leakage from surrounding normal choriocapillaris along with the yet not atrophic overlying RPE. On FAF, LC appear as hypoautofluorescent lines, but this approach has a low detection rate, maybe because of the presence of a continuous RPE-BM overlying line (19).

PCA lesions enlarge with increasing age and may also coalesce with other focal atrophic areas. Enlargement of PCA lesions depend on the location and origin, with those related to posterior staphyloma growing toward the macula, and those with onset within the macular region enlarging in all directions (6, 7, 20).

**Figure 2:**
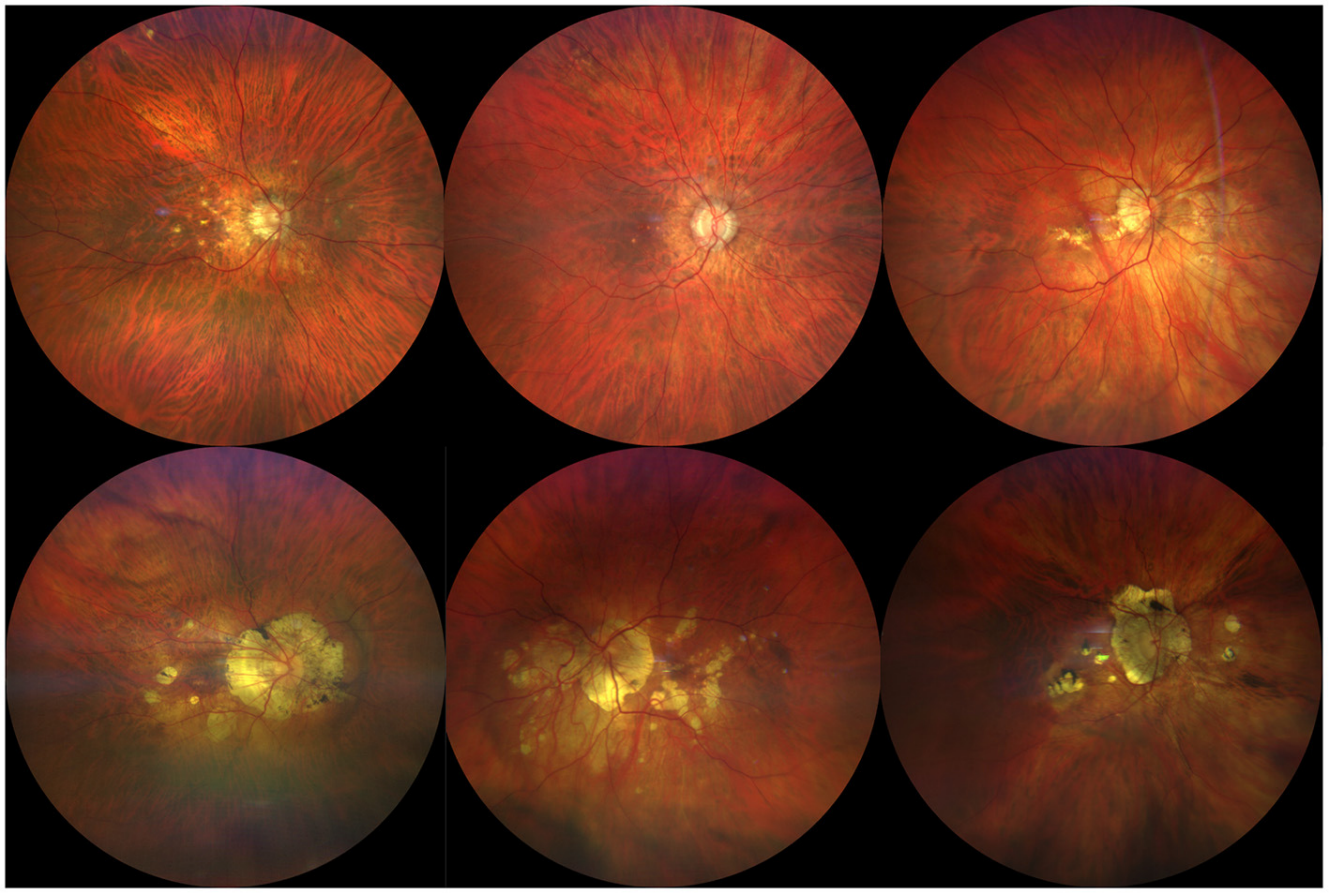
Illustrative examples of atrophic chorioretinal changes in patients with multifocal choroiditis (MFC). Top row shows cases with multifocal small rounded atrophic lesions in patients with recent diagnosis of MFC. Bottom row shows long standing MFC cases with larger and more profound chorioretinal atrophic changes associating eventual pigmentation of the margins.

**Figure 3:**
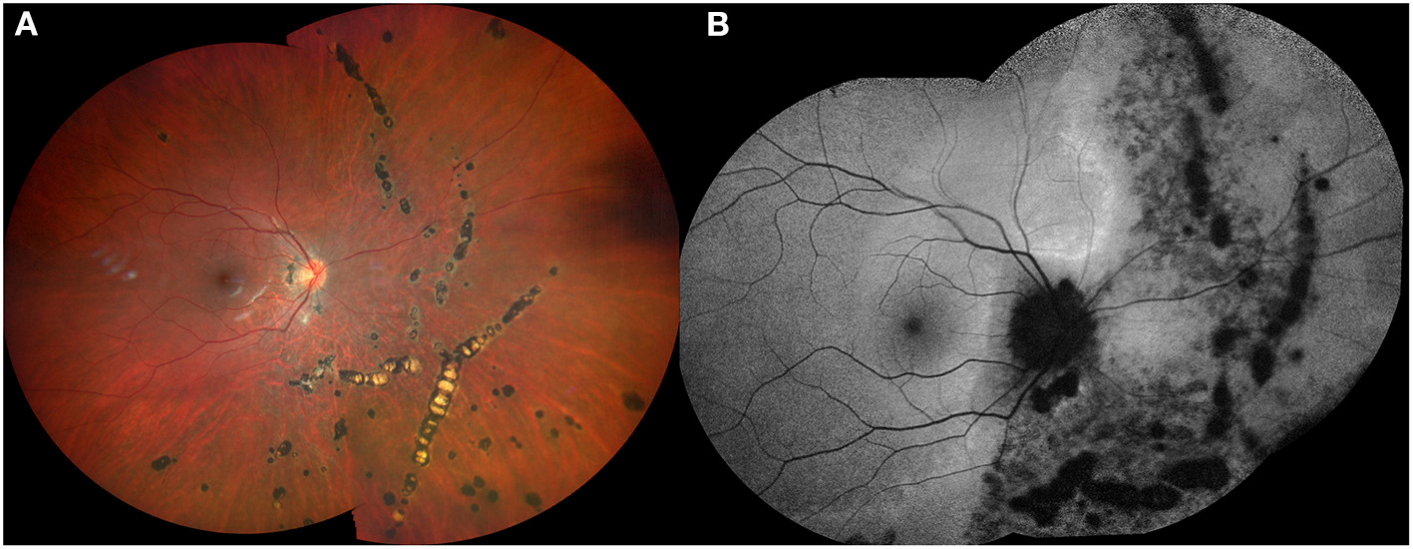
Fundus color photograph **(A)** and fundus autofluorescence image **(B)** of a 26-year-old myopic female showing curvilinear streaks in the nasal midperipheral and peripheral retina of her right eye in association with multifocal choroidits.

Chorioretinal atrophy is also included in the MFC spectrum. When acute inflammatory lesions subside, they may evolve progressively into punched-out atrophic scars, with a variable degree of pigmentation (21). Their atrophic legacy seems to depend on the degree and duration of inflammation and the resilience of the RPE, retina and choroid to the inflammatory injury. Their particular size, usually smaller than one disc area, the random distribution throughout the posterior pole and the midperipheral retina, and the lack of relationship between their location and the retinal vessels or the posterior staphyloma may be helpful to differentiate them from typical PCA and LC lesions. Also, new peripheral lesions spreading centripetally and sparing the macular area may develop with an eventual progression of the disease, leading to chorioretinal zonal or diffuse atrophy (22–24) (Figure 2). Curvilineal streaks may develop in the mid or far peripheral retina in cases with MFC, representing a hallmark feature of the disease, originally described by Nozik and Dorsch (25). This finding is commonly known as Schlaegel streaks according to the description of this author in patients with presumed ocular histoplasmosis syndrome (26). Curvilinear streaks result from coalescence of atrophic lesions along longitudinal lines (Figure 3).

## Inflammatory Chorioretinal Changes

**Figure 4:**
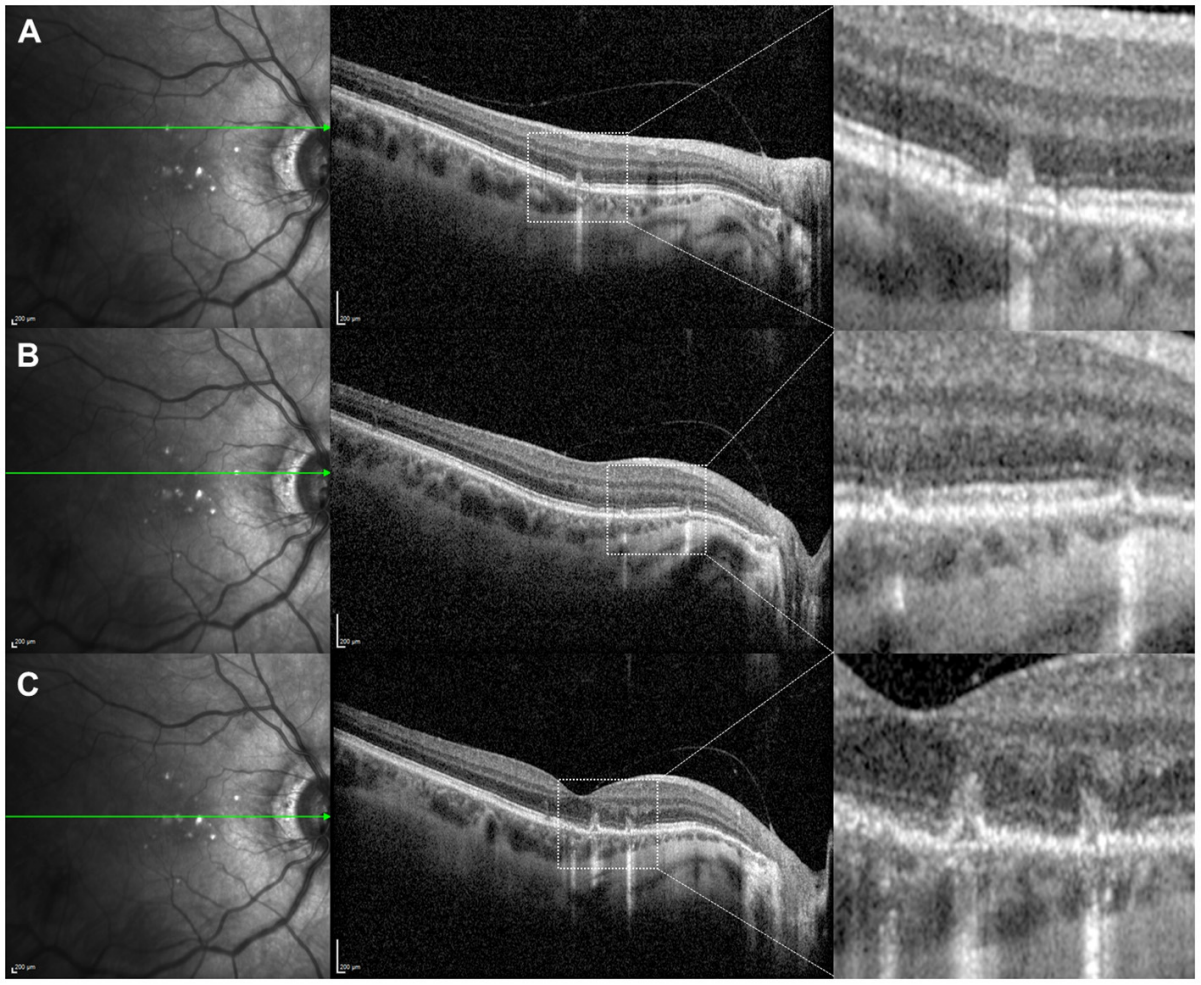
Optical coherence tomography (OCT) assessment of the macula in a 42-year-old female myopic patient complaining of scintillating scotoma in her right eye, showing typical structural changes of acute multifocal choroiditis lesions. **(A)** Superior OCT scan reveals a focal disruption of the retinal pigment epithelium (RPE) layer leading to choroidal hypertransmission, with subtle elevation of the borders; this is associated with external limiting membrane (ELM) and photoreceptors ellipsoid zone disruption, and hyperreflective subretinal and sub-RPE material. **(B)** OCT scan immediately superior to the fovea reveals two small RPE disruptions with secondary choroidal hypertransmission, and disruption of the photoreceptors ellipsoid zone with preservation of ELM, corresponding with early inflammatory lesions. **(C)** Foveal-centered OCT scan reveals two small RPE disruptions with elevation of the RPE borders, underlying choroidal hypertransmission and subretinal hyperreflective material in an area of ELM and photoreceptors ellipsoid zone disruption.

**Figure 5:**
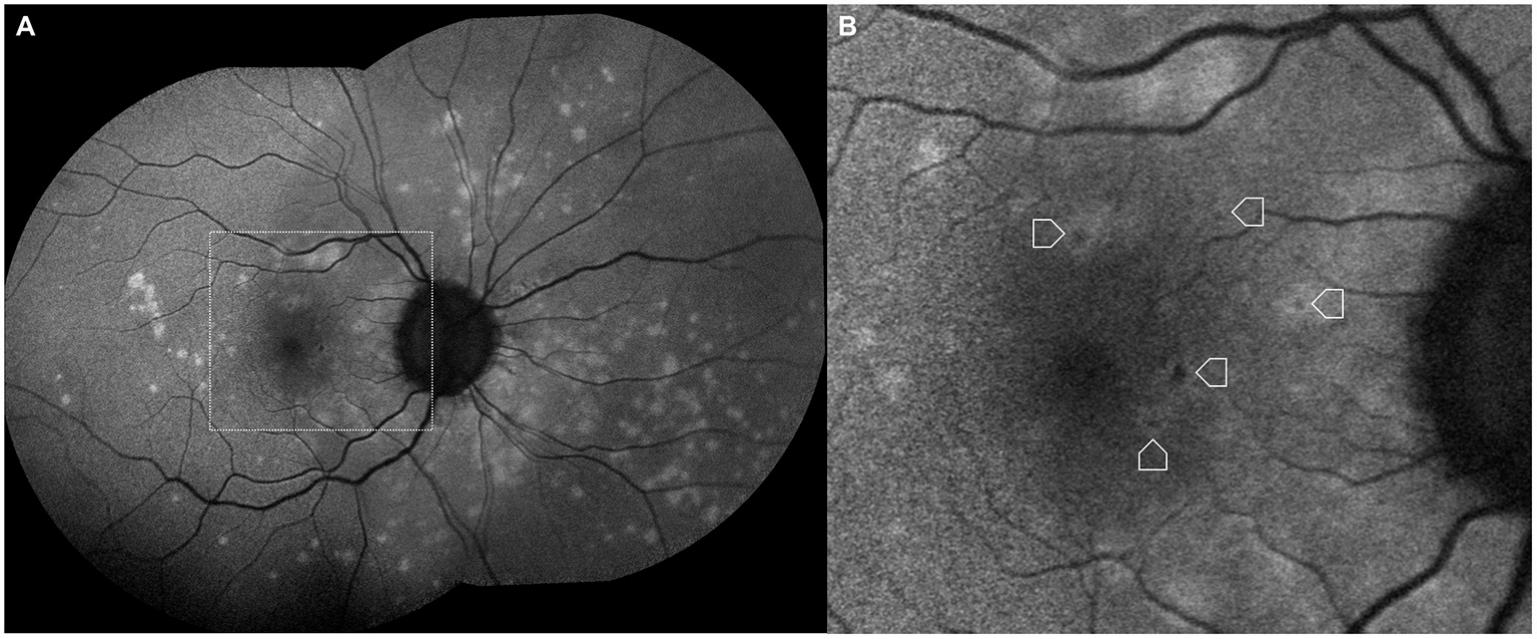
**(A)** Fundus autofluorescence image in a case of acute multifocal choroiditis showing hyperautofluorescence due to unmasking secondary to photoreceptor disruption. **(B)** Referenced detail revealing diffuse zonal hyperautofluorescence associated with small rounded hypoautofluorescent lesions corresponding to focal retinal pigment epithelium disruptions (arrowheads).

MFC acute inflammatory lesions can be observed as single or multiple yellow-grayish spots (21, 22). The typical OCT signs include (21–24): transient mild thickening of the underlying choroid; multifocal abrupt elevation of the RPE layer with disruption in the apex, leading to choroidal hypertransmission of the tomographic signal; subretinal hyperreflective material with various configurations but in close topographical relationship with the RPE changes; eventual presence of posterior vitreous inflammatory cells (Figure 4). During the acute inflammatory stage of MFC, FAF images usually evidence two distinguished features: on the one hand, concrete small hypoautofluorescent dots corresponding to the focal RPE disruptions of the lesions; on the other hand, the development of multifocal outer retinal layers disruption leads to an unmasking effect (23, 24) and faint diffuse hyperautofluorescence (Figure 5).

**Figure 6:**
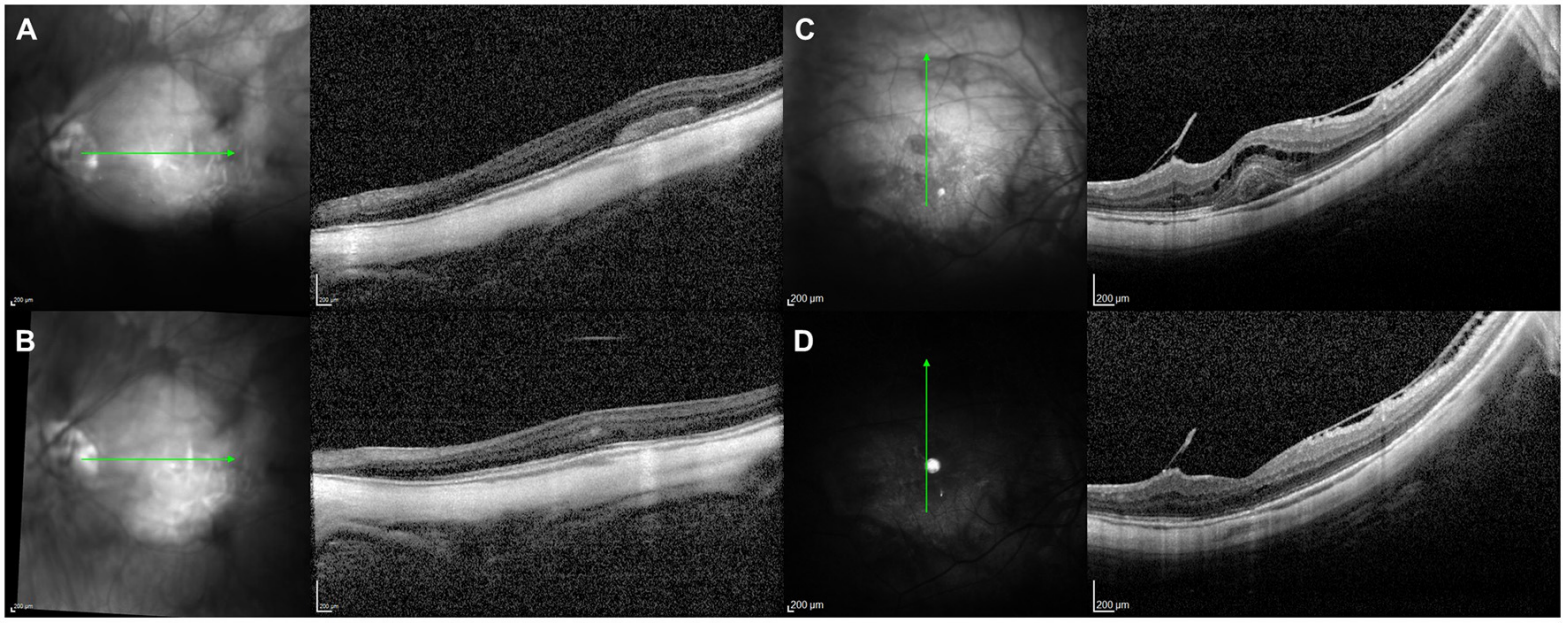
Illustrative examples of myopic spontaneous subretinal hemorrhages. **(A,C)** show high density subretinal material corresponding to hemorrhage overlying an intact layer of retinal pigment epithelium (RPE). **(B,D)** represent the corresponding images evidencing complete resolution after 8 weeks of follow-up with no evidence of subretinal scar or RPE disturbance.

Inflammatory chorioretinal changes in patients with pathologic myopia must be distinguished from other acute symptomatic structural changes not related to MFC. Myopic spontaneous subretinal hemorrhages are tomographically observed as hyperreflective subretinal material overlying an intact RPE layer (Figure 6). Subretinal bleeding usually are self-limited and absorb spontaneously with no need to perform any therapeutic approach.

## Choroidal Neovascularization

The early identification of early choroidal neovascularization (CNV) signs in patients with pathologic myopia or MFC is key to optimize the final visual outcome by prompting intravitreal injection of vascular endothelial growth factor (VEGF) inhibitors. CNV represents a leading cause of significant visual impairment in patients with pathologic myopia and MFC.

Myopic and inflammatory CNV develop as type 2 neovascular lesions, growing from the choriocapillaris through an eroded RPE into the subretinal space (27). These neovascular lesions typically lead to high density subretinal exudation and intraretinal fluid. FA shows early hyperfluorescence with subsequent leakage given the vascular nature of the subretinal lesion, suitable with a predominantly classic pattern. However, inflammatory lesions in MFC may also lead to early hyperfluorescence secondary to the window defect due to the RPE erosions described.

**Figure 7:**
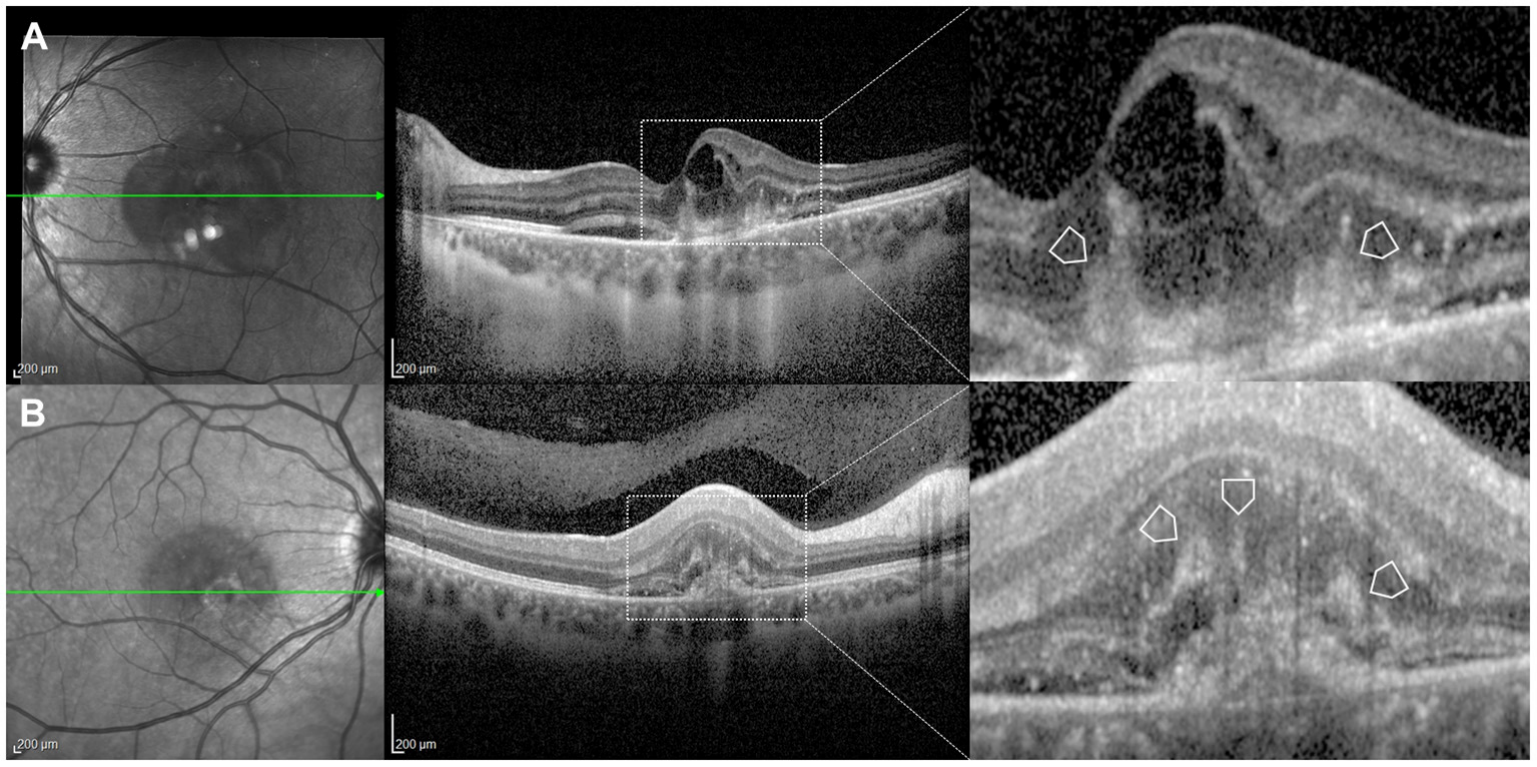
Illustrative examples of inflammatory choroidal neovascularization (CNV) in cases of a 37-year-old female **(A)** and a 25-year-old female **(B)** with multifocal choroiditis. The enlarged sections of the scans sign show typical multiple finger-like projections (arrowheads) arising from the area of CNV into the outer retinal layers. These represent the pitchfork sign as described by Hoang et al. (28).

**Figure 8:**
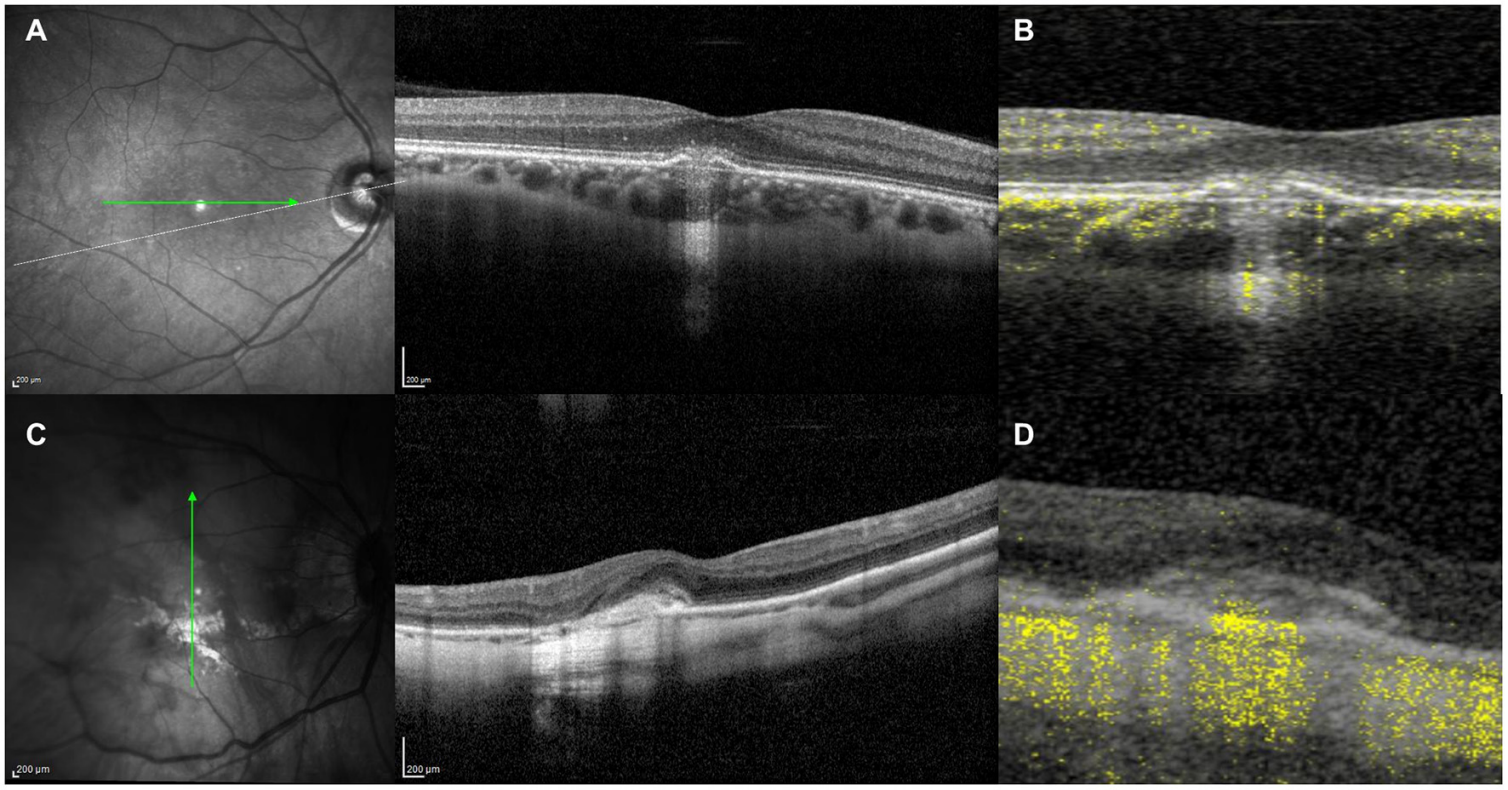
**(A)** Structural optical coherence tomography (OCT) scan showing typical signs of an acute inflammatory lesion in a 41-year-old myopic female with central vision loss including focal retinal pigment epithelium (RPE) disruption with elevation of the borders leading to underlying choroidal hypertransmission, associated with subretinal hyperreflective material and disruption of the overlying external limiting membrane (ELM) and photoreceptors ellipsoid zone. **(B)** Structural OCT scan with flow signal overlay as analyzed by OCT-Angiography (OCT-A) ruling out the presence of flow signal within the inflammatory lesion, which allows to discard the presence of choroidal neovascularization. **(C)** Structural OCT scan showing signs of exudation with high-density subretinal material in a 52-year-old myopic female with previous history of multifocal choroditis. **(D)** Structural OCT scan with flow signal overlay as analyzed by OCT-A confirming the presence of flow signal within the subretinal hyperreflective tissue, suggestive of choroidal neovascularization.

Thus, the differentiation of neovascularization and inflammation by FA might be challenging (21). The pitchfork sign is a distinctive finding in CNV with inflammatory etiology (28), described as the presence in OCT structural images of multiple finger-like projections arising from the area of CNV into the outer retina (Figure 7). Optical coherence tomography angiography (OCT-A) is an essential tool to differentiate accurately between inflammation and neovascularization in cases with subretinal exudation (Figure 8) by ruling out or confirming the presence of flow signal within the structural abnormality (21, 29).

## Overlap Between Pathologic Myopia and Multifocal Choroditis

**Figure 9:**
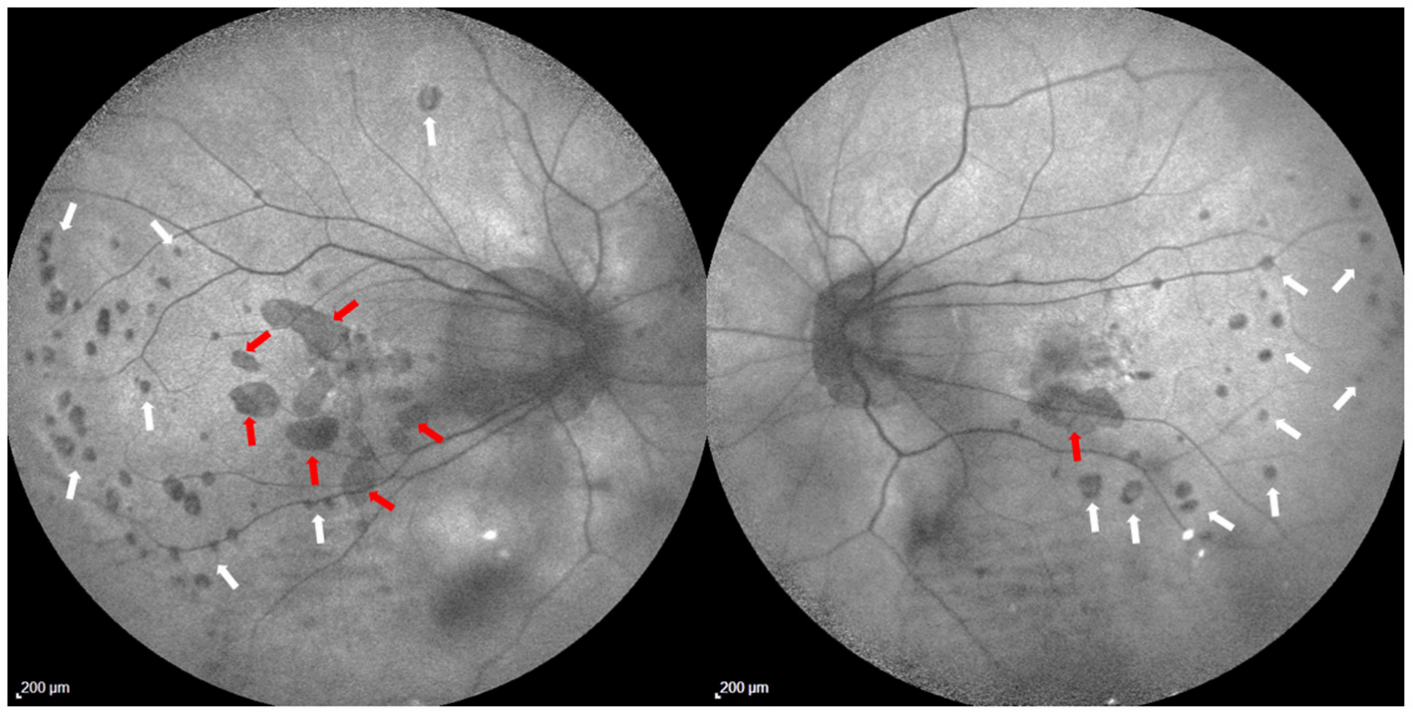
Fundus autofluorescence images in a 46-year-old female with high myopia and history of multifocal choroiditis. Two different atrophic patterns can be identified: patchy chorioretinal atrophic lesions (red arrows) are related to pathologic myopia and located within the posterior pole and the peripapillary area; small rounded hypoautofluorescent spots (white arrowheads) are related to atrophic multifocal changes due to multifocal choroditis, and a faint hyperautofluorescence (unmasking effect) can be observed surrounding these lesions in both eyes. A typical curvilinear distribution of these hypoautofluorescent spots is evident in the temporal region of both eyes.

MFC is more common in myopic female patients. This opens the possibility to find structural fundus changes related to both the refractive condition and the inflammatory disorder. The overlap between atrophic myopic and MFC is more frequent in patients after the fifth decade of life that may develop chorioretinal atrophic changes in association with previous MFC healed lesions (Figure 9).

## Therapeutic Approach

**Figure 10:**
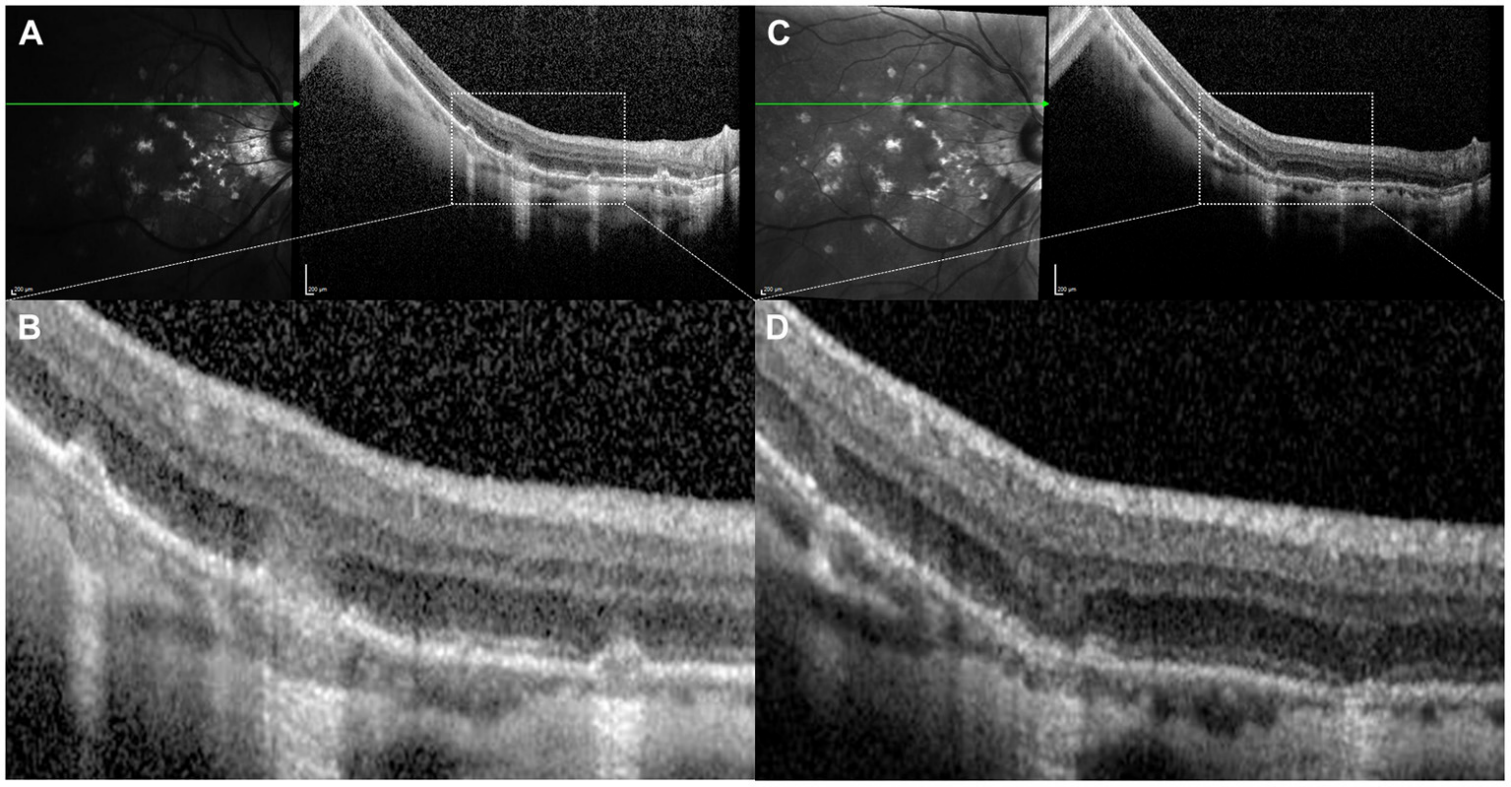
Optical coherence tomography (OCT) scans in a 49-year-old male myopic patient with recurrent inflammation due to multifocal choroidits. **(A)** OCT scan coincident with acute symptoms showing multifocal areas of disruption of the retinal pigment epithelium (RPE) layer leading to choroidal hypertransmission. **(B)** Detail of the previous image revealing structural lesions consisting on RPE disruption with secondary choroidal hypertransmision, associated with overlying external limiting membrane (ELM) and photoreceptors ellipsoid zone disruption, and hyperreflective subretinal material. **(C)** OCT scan performed after 3 weeks of oral steroids treatment showing decrease in choroidal thickness, regression of hyperreflective material and suboptimal restoration of the RPE layer. **(D)** Detail of the previous image revealing significant decrease in choroidal thickness compared with the baseline image **(B)** and decrease in choroidal hypertransmission due to suboptimal restoration of the RPE layer. There is complete regression of the subretinal hyperreflective material and persistence of MLE and photoreceptors ellipsoid zone disruption.

There is a significant lack of evidence-based guidelines for the management of the inflammatory activity of MFC cases. Oral prednisone at doses >10 mg/day have been proven to decrease the risk of structural complications (30) so in the presence of vision threatening complications this approach should be considered. The response is typically rapid in terms of symptoms improvement and structural recovery of lesions (Figure 10). However, as in many other intraocular inflammations, the prolonged and repeated use of steroids is associated with a significant risk of secondary effects. Therefore, the use of immunosuppressant steroid-sparing agents should be considered in the presence of inflammatory recurrences with visual threatening. The most commonly one used is mycophenolate as mofetil (CellCept, Roche, Basel, Switzerland) or sodium (Myfortic, Novartis, Basel, Switzerland) although no randomized clinical trial has been performed (31, 32). Thus, the dosage and duration of this treatment is completely empirical, but the general reported use coincides overall in doses of 500–1,000 mg twice per day (mofetil), or 360–720 mg twice per day (sodium).

On the other hand, inflammatory and myopic CNV need intravitreal anti-VEGF drugs to induce regression of exudative manifestations related to the neovascular process. The general consensus about the protocol to use with anti-VEGF in inflammatory CNV indicates to perform a first intravitreal treatment followed by observation afterwards. The addition of intraocular or periocular steroid injection might not be helpful to improve the response or the visual prognosis compared with monotherapy with anti-VEGF, but instead increasing the adverse events with intraocular pressure rise or cataract formation.

## Conclusion

Atrophic or neovascular changes present in myopic patients should always include MFC as a potential underlying etiology. The number, shape and distribution of atrophic lesions makes possible to distinguish PM and MFC in a conventional fundus examination, but multimodal analysis with OCT and FAF imaging may add valuable information to perform such distinction. The sudden onset of visual loss and complaint might be related to inflammatory events, choroidal neovascularization or spontaneous subretinal hemorrhage. The present manuscript details the most relevant structural changes that OCT and FAF can highlight related to these distinct processes. The management, prognosis and follow-up schedule should be adapted to a comprehensive and thoughtful approach in patients with atrophic or neovascular lesions, considering MFC as a potential underlying diagnosis in patients with myopia.

## Author Contributions

All authors listed have made a substantial, direct, and intellectual contribution to the work and approved it for publication.

## Conflict of Interest

RG-P is a consultant to Carl Zeiss Meditec, Novartis, ORA Clinical, and Roche. RD-M is a consultant to Heidelberg Engineering, Novartis, and Roche. The remaining author declares that the research was conducted in the absence of any commercial or financial relationships that could be construed as a potential conflict of interest.

## Publisher's Note

All claims expressed in this article are solely those of the authors and do not necessarily represent those of their affiliated organizations, or those of the publisher, the editors and the reviewers. Any product that may be evaluated in this article, or claim that may be made by its manufacturer, is not guaranteed or endorsed by the publisher.
